# Supplementary material for: Prokaryote Distribution Patterns along a Dissolved Oxygen Gradient Section in the Tropical Pacific Ocean
Source: Microorganisms. 2023 Aug 28;11(9):2172. doi: 10.3390/microorganisms11092172 (PMC10534896; doi:10.3390/microorganisms11092172)
Supplement: Supplementary file 1 [file microorganisms-11-02172-s001.zip › Supplementary Table S1.pdf]

Supplementary Table S1. Sampling station and depth details.

Station	Sample Depth (m)
V2114	10, 30, 50, 75, 100, 150, 200, 300, 500, 800, 1000, 1250, 2000, 2500, 3000, 3500, 4000, 5000, 5500, 5900
V1713	10, 30, 50, 75, 100, 150, 200, 300, 500, 800, 1000, 1250, 1500, 2000, 2500, 3500, 4000, 4500, 5000
V1312	10, 30, 50, 75, 100, 150, 200, 300, 500, 800, 1000, 1250, 1500, 2000, 2500, 3000, 3500, 4000, 4630
V0605	10, 30, 50, 75, 100, 150, 200, 300, 500, 800, 1000, 1250, 1500, 2000, 2500, 3000, 3500, 4000, 4500
IV1713	10, 30, 50, 75, 100, 150, 200, 300, 500, 800, 1000, 1250, 1500, 2000, 2500, 3000, 3500, 4000
IV2016	10, 30, 50, 75, 100, 150, 200, 300, 500, 800, 1000, 1250, 1500, 2000, 2500, 3000, 3500, 4000, 4500
