# Supplementary figures and images for: Prokaryote Distribution Patterns along a Dissolved Oxygen Gradient Section in the Tropical Pacific Ocean
Source: Microorganisms. 2023 Aug 28;11(9):2172. doi: 10.3390/microorganisms11092172 (PMC10534896; doi:10.3390/microorganisms11092172)

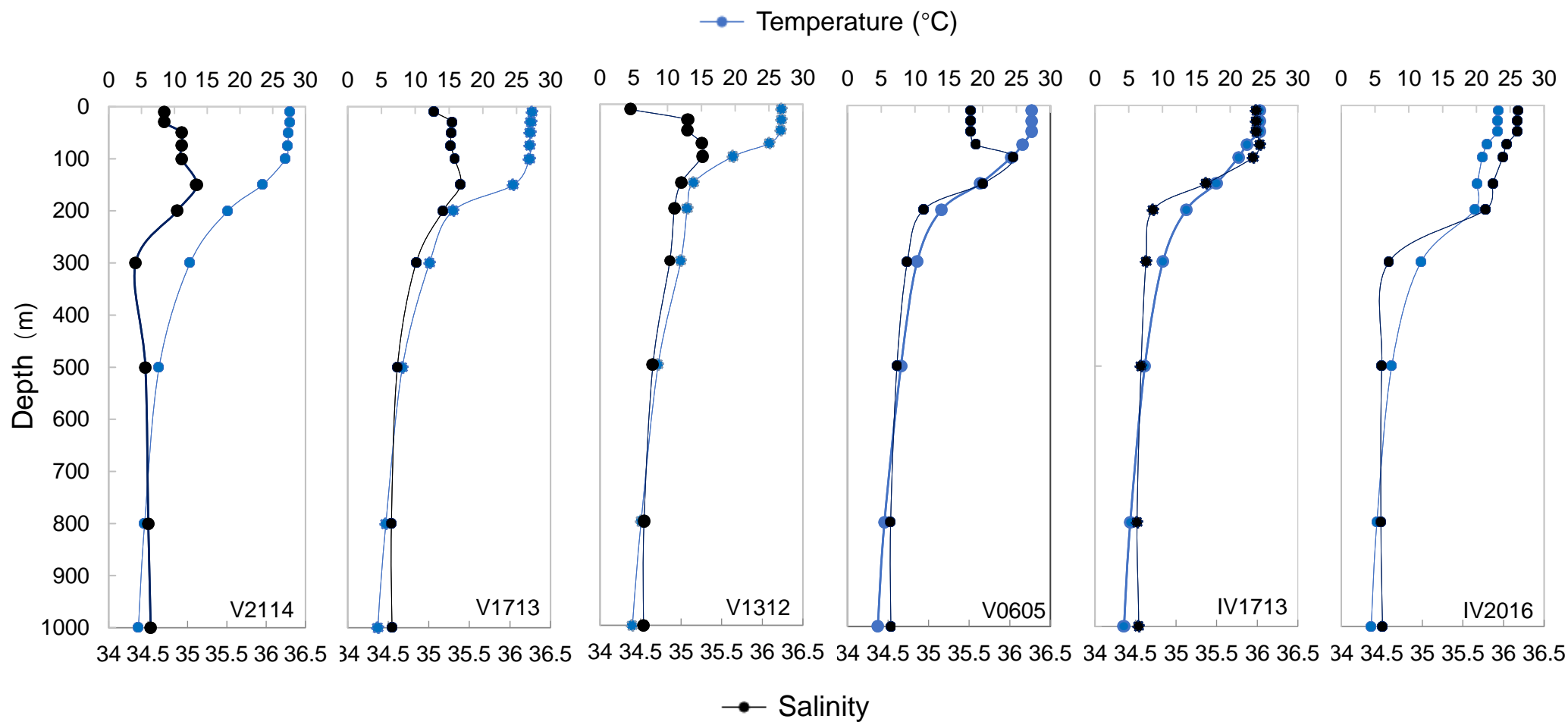

Supplemental Figure S1 The temperature and salinity profiles along the water columns ( $\leq 1000$  m)

Supplement: Supplementary file 1 [file microorganisms-11-02172-s001.zip › Supplementary Figure S1.pdf]

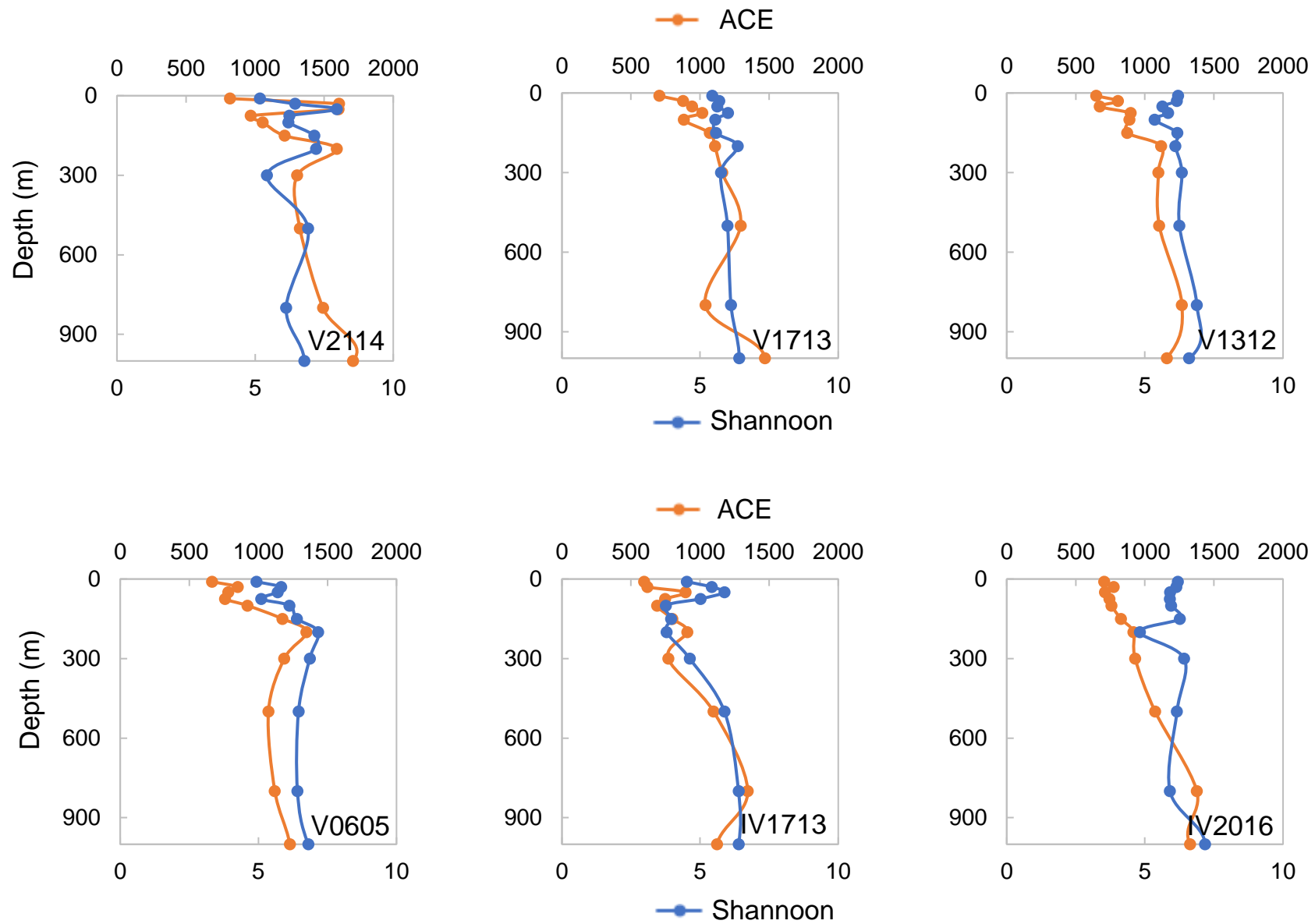

Supplemental Figure S2 Alpha diversity indices (ACE and Shannon) along the water columns ( $\leq 1000$  m)

Supplement: Supplementary file 1 [file microorganisms-11-02172-s001.zip › Supplementary Figure S2.pdf]
